# COVID-19 Outcomes Stratified by Control Status of Hypertension and Diabetes: Preliminary Findings From PCORnet, U.S.

**DOI:** 10.1016/j.focus.2022.100012

**Published:** 2022-07-10

**Authors:** Sandra L. Jackson, Jason P. Block, Deborah B. Rolka, Meda E. Pavkov, Jennifer R. Chevinsky, Akaki Lekiachvili, Thomas W. Carton, Deepika Thacker, Joshua L. Denson, Anuradha Paranjape, Michael D. Kappelman, Tegan K. Boehmer, Evelyn Twentyman

**Affiliations:** 1 Division for Heart Disease and Stroke Prevention, National Center for Chronic Disease Prevention and Health Promotion (NCCDPHP), Centers for Disease Control and Prevention, Atlanta, Georgia; 2 Division of Chronic Disease Research Across the Lifecourse (CoRAL), Department of Population Medicine, Harvard Pilgrim Health Care Institute, Harvard Medical School, Boston, Massachusetts; 3 Division for Diabetes Translation, National Center for Chronic Disease Prevention and Health Promotion (NCCDPHP), Centers for Disease Control and Prevention, Atlanta, Georgia; 4 Division of Nutrition, Physical Activity, and Obesity, National Center for Chronic Disease Prevention and Health Promotion (NCCDPHP), Centers for Disease Control and Prevention, Atlanta, Georgia; 5 National Center for Chronic Disease Prevention and Health Promotion (NCCDPHP), Centers for Disease Control and Prevention, Atlanta, Georgia; 6 Louisiana Public Health Institute, New Orleans, Louisiana; 7 Nemours Cardiac Center, Nemours Children's Health, Jacksonville, Florida; 8 Section of Pulmonary, Critical Care, and Environmental Medicine, Tulane University School of Medicine, New Orleans, Louisiana; 9 Temple University, Philadelphia, Pennsylvania; 10 Department of Pediatrics, UNC School of Medicine, The University of North Carolina at Chapel Hill, Chapel Hill, North Carolina; 11 Center for Surveillance, Epidemiology, and Laboratory Services (CSELS), Centers for Disease Control and Prevention, Atlanta, Georgia

**Keywords:** Hypertension, diabetes mellitus, COVID-19, glycated hemoglobin, blood pressure

## Abstract

**Introduction:**

Hypertension and diabetes are associated with increased COVID-19 severity, yet less is known about COVID-19 outcomes across levels of disease control for these conditions.

**Methods:**

All adults aged ≥20 years with COVID-19 between March 1, 2020 and March 15, 2021 in 42 healthcare systems in National Patient-Centered Clinical Research Network were identified.

**Results:**

Among 656,049 adults with COVID-19, 41% had hypertension, and 13% had diabetes. Of patients with classifiable hypertension, 35% had blood pressure <130/80 mmHg, 40% had blood pressure of 130‒139/80‒89 mmHg, 21% had blood pressure of 140‒159/90‒99 mmHg, and 6% had blood pressure ≥160/100 mmHg. Severe COVID-19 outcomes were more prevalent among those with blood pressure of ≥160/100 than among those with blood pressure of 130–139/80–89, including hospitalization (23.7% [95% CI=23.0, 24.4] vs 11.7% [95% CI=11.5, 11.9]), receipt of critical care (5.5% [95% CI=5.0, 5.8] vs 2.4% [95% CI=2.3, 2.5]), receipt of mechanical ventilation (3.0% [95% CI=2.7, 3.3] vs 1.2% [95% CI=1.1, 1.3]), and 60-day mortality (4.6% [95% CI=4.2, 4.9] vs 1.8% [95% CI=1.7, 1.9]). Of patients with classifiable diabetes, 44% had HbA1c <7%, 35% had HbA1c 7% to <9%, and 21% had HbA1c ≥9%. Hospitalization prevalence was 31.3% (95% CI=30.7, 31.9) among those with HbA1c <7% vs 40.2% (95% CI=39.4, 41.1) among those with HbA1c ≥9%; other outcomes did not differ substantially by HbA1c.

**Conclusions:**

These findings highlight the importance of appropriate management of hypertension and diabetes, including during public health emergencies such as the COVID-19 pandemic.

## HIGHLIGHTS

• Among 656,049 adults with COVID-19, 41% had hypertension, and 13% had diabetes.
• Approximately one quarter of those with hypertension had blood pressure >140/90.
• Approximately one fifth of those with diabetes had HbA1c >9%.
• Worse COVID-19 outcomes were more prevalent among those with higher blood pressure.
• Hospitalization was more prevalent among those with higher HbA1c.

## Graphical abstract

**Figure:**
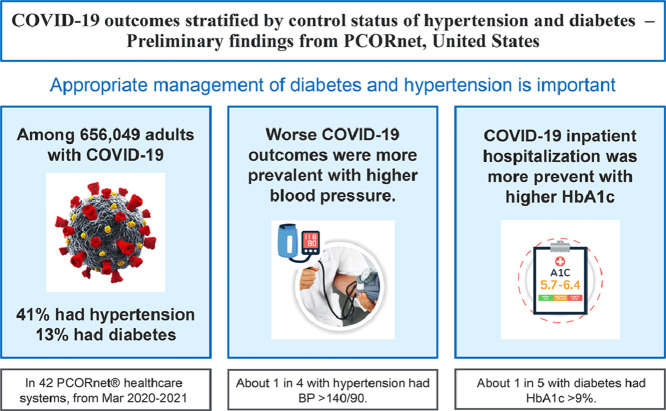

## INTRODUCTION

Hypertension and diabetes are 2 of the most common comorbidities among patients hospitalized with coronavirus disease 2019 (COVID-19) infection^1^ and have been associated with more severe COVID-19 and mortality.^2^, ^3^, ^4^ Poor management of chronic diseases increases cardiovascular morbidity and mortality^5^ and may be associated with worse COVID-19 outcomes.^6^, ^7^, ^8^ Furthermore, existing racial and ethnic disparities in the prevalence and control of chronic diseases^5^ might exacerbate disparities in COVID-19 outcomes.^9^ The purpose of this study was to describe the demographic and clinical characteristics and COVID-19 outcomes across strata of disease control for adults with hypertension and diabetes.

## METHODS

We aggregated outpatient, emergency department, and inpatient electronic health record data in 42 healthcare systems participating in National Patient-Centered Clinical Research Network (PCORnet)^10^^,^^11^ to identify all adults aged ≥20 years with COVID-19, identified by positive severe acute respiratory syndrome coronavirus 2 (SARS-CoV-2) polymerase chain reaction (99%) or antigen (1%) laboratory test, between March 1, 2020 and March 15, 2021.

Hypertension and diabetes were identified using PCORnet electronic health record documentation from the previous 3 years. *Hypertension* was defined on the basis of (1) prescription for an outpatient antihypertensive medication (not including centrally acting agents, loop diuretics, or beta-blockers because these are commonly used for other conditions); (2) outpatient centrally acting agents, loop diuretics, or β blockers and either an ICD-10-CM diagnostic code for hypertension or outpatient blood pressure (BP) ≥130/80 mmHg; (3) 2 outpatient BP readings ≥130/80 mmHg on separate days; or (4) 2 ICD-10-CM codes for hypertension on separate days in any care setting. *Diabetes* was defined as (1) a prescription for an outpatient diabetes medication, (2) HbA1c ≥6.5%, (3) 1 ICD-10-CM code for type 1 diabetes or type 2 diabetes plus metformin, or (4) 2 ICD-10-CM codes for diabetes in any care setting.

Disease control status was identified by the measurement (HbA1c or outpatient BP) most recently available before the COVID-19‒positive laboratory test. BP values within 2 weeks before the COVID-19 event were excluded to avoid BP values during the acute illness phase. Up to an 18-month lookback period was used to assess disease control to allow most patients to have had an outpatient visit; patients without an HbA1c or BP value or only with values beyond 18 months before the COVID-19 positive laboratory test were not classified into disease control status categories for diabetes or hypertension, respectively. Hypertension control was categorized into 4 strata (BP <130/80 mmHg, 130‒139/80‒89 mmHg, 140‒159/90‒99 mmHg, or ≥160/100 mmHg) using the higher systolic or diastolic value if a patient's values fell into multiple levels. Diabetes control was categorized into 3 strata (HbA1c <7%, 7 to <9%, ≥9%). Collected patient demographic and clinical characteristics included age, sex, race (White, Black, Asian, other), Hispanic ethnicity, overweight or obese BMI (≥25 kg/m^2^), COVID-19 treatments (dexamethasone, other corticosteroids, remdesivir), and outcomes (hospitalization, receipt of critical care, receipt of mechanical ventilation, and 60-day mortality). All analyses were descriptive in nature; analyses were conducted in 2021 using patient-level data at individual PCORnet sites and then aggregated into this report. Patient-level statistical tests could not be conducted because only summary data were aggregated from individual sites; methods are being developed to conduct pooled regression analyses in the distributed PCORnet environment. This secondary data analysis of aggregate data in support of the federal COVID-19 emergency response was considered not to be human subjects research and was exempt from IRB review.

## RESULTS

Among 656,049 adults with COVID-19; 54% were female, 62% were White, 15% were Black, and 3% were Asian (10% missing race); 18% were Hispanic, and 71% were non-Hispanic (9% missing ethnicity); and 76% had overweight or obese BMI. A small percentage were treated with dexamethasone (6%), other corticosteroids (5%), or remdesivir (5%), and among all patients with COVID-19, 14% were hospitalized, 3% received critical care, 2% received mechanical ventilation, and 2% had 60-day mortality.

**Figure 1:**
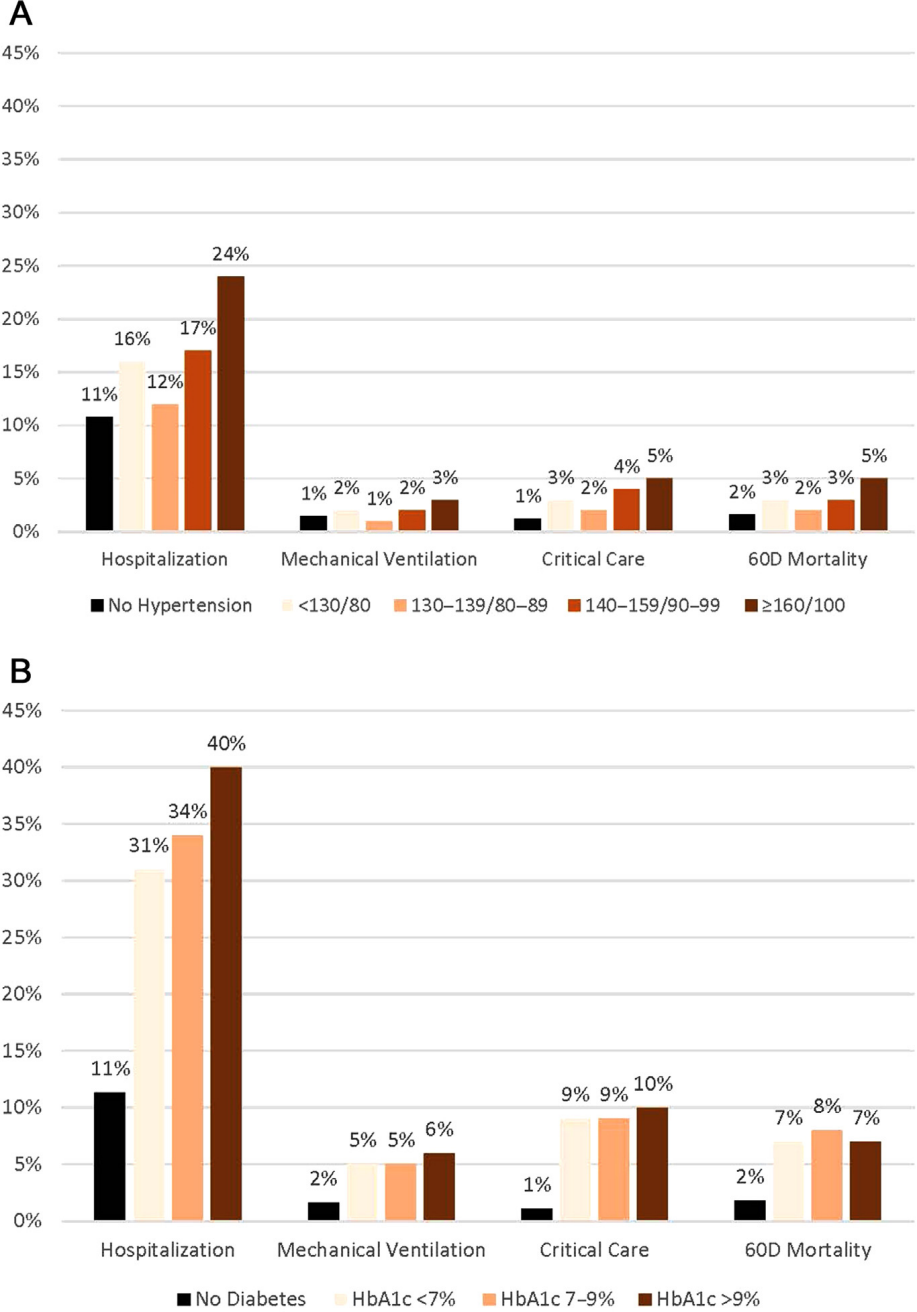
COVID-19 outcomes by (A) hypertension control status and (B) diabetes control status. This figure shows variation in inpatient hospitalization, mechanical ventilation, critical care use, and 60D mortality by strata of chronic disease control. For *no hypertension, n*=389,101, and for *no diabetes, n*=571,323. For the entire cohort, N=656,049, and outcome prevalences were 14% inpatient hospitalization, 2% mechanical ventilation, 2% critical care, and 3% 60D mortality. 60D, 60-day.

Overall, 266,948 (41%) patients with COVID-19 met our criteria for hypertension. Of 209,293 patients whose hypertension control could be classified (78%), 35% had BP <130/80 mmHg, 40% had BP of 130–139/80–89 mmHg, 21% had BP of 140–159/90–99 mmHg, and 6% had BP ≥160/100 mmHg (Table 1). Black persons were disproportionately represented in the highest BP group (25.0%; 95% CI=24.2, 25.7) compared with representation in the lowest BP group (14.1%; 95% CI=13.8, 14.3). Severe COVID-19 outcomes generally increased in frequency across the upper 3 categories of BP control. Among adults with BP of 130–139/80–89 mmHg compared with those with BP of ≥160/100 mmHg, hospitalization was 11.7% (95% CI=11.5, 11.9) vs 23.7% (95% CI=23.0, 24.4), receipt of critical care was 2.4% (95% CI=2.3, 2.5) vs 5.4% (95% CI=5.0, 5.8), mechanical ventilation was 1.2% (95% CI=1.1, 1.3) vs 3.0% (95% CI=2.7, 3.3), and 60-day mortality was 1.8% (95% CI=1.7, 1.9) vs 4.6% (95% CI=4.2, 4.9) (Figure 1A).

**Table 1:** Demographic and Clinical Characteristics of Patients With COVID-19 by Hypertension Control Status

Characteristics	All with COVID-19	All HTN,^a^ % (95% CI)	BP control category,^b^ % (95% CI)			
			<130/80	130‒139/80‒89	140‒159/90‒99	≥160/100
	656,049	266,948	73,618	77,703	44,884	13,088
Overall	100%	40.7 (40.5, 40.9)	35.2 (34.8, 35.5)	39.6 (39.3, 39.9)	21.4 (21.1, 21.8)	6.3 (5.8, 6.7)
Age, years						
20–39	38.6 (38.5, 38.7)	21.1 (21.0, 21.3)	26.0 (25.6, 26.3)	25.7 (25.4, 26.1)	17.3 (17, 17.7)	12.0 (11.5, 12.6)
40–54	26.0 (25.9, 26.1)	25.9 (25.7, 26.0)	23.8 (23.5, 24.1)	29.7 (29.4, 30.0)	27.1 (26.7, 27.5)	26.2 (25.4, 26.9)
55–64	16.0 (15.9, 16.1)	21.5 (21.4, 21.7)	20.2 (19.9, 20.5)	21.3 (21, 21.6)	23.2 (22.8, 23.6)	23.8 (23.0, 24.5)
65–74	10.9 (10.8, 11.0)	17.2 (17.1, 17.4)	16.8 (16.5, 17.1)	14.4 (14.1, 14.6)	18.6 (18.3, 19.0)	20.3 (19.6, 21.0)
75–84	5.9 (5.8, 5.9)	9.9 (9.8, 10.0)	9.6 (9.4, 9.8)	6.7 (6.5, 6.9)	10.0 (9.7, 10.3)	12.7 (12.1, 13.3)
≥85	2.6 (2.6, 2.7)	4.3 (4.3, 4.4)	3.7 (3.6, 3.9)	2.2 (2.1, 2.3)	3.7 (3.5, 3.9)	5.0 (4.7, 5.4)
**Sex**						
Female	54.2 (54.1, 54.3)	55.1 (54.9, 55.3)	61.7 (61.3, 62.0)	55.9 (55.6, 56.3)	51.2 (50.8, 51.7)	51.0 (50.2, 51.9)
Male	45.7 (45.6, 45.9)	44.9 (44.7, 45.1)	38.3 (38, 38.7)	44.1 (43.7, 44.4)	48.8 (48.3, 49.2)	49.0 (48.1, 49.8)
Hispanic ethnicity^c^						
Yes	18.4 (18.3, 18.5)	15.3 (15.2, 15.5)	15.2 (15, 15.5)	14.8 (14.6, 15.1)	14.8 (14.5, 15.1)	15.4 (14.8, 16.0)
No	71.2 (71.1, 71.4)	81.1 (81.0, 81.3)	82.6 (82.4, 82.9)	82.8 (82.5, 83)	82.8 (82.4, 83.1)	82.1 (81.4, 82.7)
Race^c^						
Asian	2.8 (2.8, 2.9)	2.4 (2.4, 2.5)	2.3 (2.2, 2.4)	2.3 (2.2, 2.4)	2.1 (2.0, 2.2)	2.2 (2.0, 2.5)
Black	14.6 (14.5, 14.7)	17.8 (17.7, 18.0)	14.1 (13.8, 14.3)	15.2 (15.0, 15.5)	18.5 (18.1, 18.9)	25.0 (24.2, 25.7)
White	61.8 (61.6, 61.9)	67.1 (67.0, 67.3)	72.6 (72.3, 73)	71.8 (71.5, 72.1)	68.3 (67.8, 68.7)	59.8 (58.9, 60.6)
Other^d^	11.1 (11.1, 11.2)	8.4 (8.3, 8.5)	7.2 (7.1, 7.4)	6.6 (6.4, 6.8)	7.2 (7.0, 7.5)	8.6 (8.2, 9.1)
BMI^c^, kg/m^2^						
<18.5	1.6 (1.6, 1.7)	1.5 (1.4, 1.4)	1.6 (1.5, 1.7)	0.9 (0.8, 1)	1.1 (1.0, 1.2)	1.8 (1.6, 2.1)
18.5 to <25	21.9 (21.8, 22.0)	16.9 (16.8, 17.1)	20.3 (20, 20.6)	13.8 (13.5, 14)	13.7 (13.4, 14.0)	15.6 (15, 16.2)
25 to <30	30.1 (30.0, 30.3)	28.6 (28.4, 28.7)	29.7 (29.4, 30.1)	27.8 (27.5, 28.2)	27.7 (27.2, 28.1)	26.6 (25.8, 27.3)
30 to <35	22.8 (22.7, 22.9)	24.8 (24.6, 24.9)	23.9 (23.5, 24.2)	26.4 (26.1, 26.7)	26.3 (25.9, 26.7)	23.9 (23.2, 24.7)
35 to <40	12.5 (12.4, 12.6)	14.6 (14.5, 14.8)	13.2 (13, 13.5)	16.3 (16.1, 16.6)	15.7 (15.4, 16.0)	15.2 (14.6, 15.8)
≥40	11.0 (10.9, 11.1)	13.6 (13.5, 13.7)	11.3 (11, 11.5)	14.8 (14.5, 15)	15.5 (15.2, 15.8)	16.9 (16.2, 17.5)
COVID-19 medications						
Dexamethasone	6.4 (6.3, 6.4)	9.6 (9.5, 9.7)	8.1 (7.9, 8.3)	6.6 (6.4, 6.8)	9.0 (8.8, 9.3)	11.1 (10.5, 11.6)
Other corticosteroids	5.4 (5.3, 5.4)	8.7 (8.6, 8.8)	8.3 (8.1, 8.5)	7.4 (7.2, 7.6)	8.8 (8.5, 9.1)	10.0 (9.5, 10.5)
Remdesivir	4.6 (4.5, 4.6)	7.4 (7.3, 7.5)	6.1 (5.9, 6.3)	4.7 (4.6, 4.9)	7.0 (6.8, 7.3)	8.9 (8.4, 9.4)

a Includes 57,655 who met the criteria for hypertension but could not be classified by control status (i.e., did not have a BP measurement within 18 months before COVID-19).

b BP control level was assessed among 209,293 persons, with BP categorized by the higher value of systolic or diastolic blood pressure, if a patient's values fell into multiple levels.

c Missing data not shown.

d Other includes PCORnet values of Native Hawaiian or other Pacific Islander, American Indian or Alaska Native, multiple race, and other. BP, blood pressure; HTN, hypertension; PCORnet, National Patient-Centered Clinical Research Network

Of patients with COVID-19, 84,726 (13%) met our criteria for diabetes. Among 56,745 whose control status could be classified (67%), 44% had HbA1c <7%, 35% had HbA1c 7% to <9%, and 21% had HbA1c ≥9% (Table 2). Hispanic persons (29.5% [95% CI=28.7, 30.3] vs 17.7% [95% CI=17.2, 8.2]) and Black persons (28.6% [95% CI=27.8, 29.4] vs 23.4% [95% CI=22.9, 24.0]) were disproportionately represented in the HbA1c ≥9% group compared with representation in the HbA1c <7% group. The frequency of hospitalization increased with the level of HbA1c, ranging from 31.3% (95% CI=30.7, 31.9) among those with HbA1c <7% to 40.2% (95% CI=39.4, 41.1) among those with HbA1c ≥9%; other outcomes did not differ substantially by HbA1c: critical care (9.0% [95% CI=8.6, 9.4] vs 10.4% [95% CI=9.8, 10.9]), mechanical ventilation (4.8% [95% CI=4.5, 5.0] vs 6.1% [95% CI=5.6, 6.5]), 60-day mortality (7.3% [95% CI=7.0, 7.6] vs 6.7% [95% CI=6.2, 7.1]) for HbA1c <7% and HbAlc ≥9% groups, respectively (Figure 1B).

**Table 2:** Demographic and Clinical Characteristics of Patients With COVID-19 by Diabetes Control Status

Characteristics		All DM^a^	HbA1c control category (*n*=56,745)		
	All with COVID-19		<7%	7%–9%	>9%
	656,049	84,726	24,900	19,930	11,915
Overall	100%	12.9 (12.7, 13.1)	43.9 (43.3, 44.5)	35.1 (34.5, 35.8)	21 (20.3, 21.7)
Age, years					
20–39	38.6 (38.5, 38.7)	9.8 (9.6, 10.0)	9.3 (8.9, 9.6)	6.4 (6.0, 6.7)	13.6 (13.0, 14.2)
40–54	26.0 (25.9, 26.1)	23.6 (23.3, 23.8)	21.6 (21.1, 22.1)	21.8 (21.2, 22.3)	32.3 (31.5, 33.2)
55–64	16.0 (15.9, 16.1)	25.6 (25.3, 25.9)	24.3 (23.8, 24.8)	26.7 (26.1, 27.4)	27.9 (27.1, 28.7)
65–74	10.9 (10.8, 11.0)	23.2 (22.9, 23.5)	25.3 (24.7, 25.8)	25.8 (25.2, 26.4)	17.0 (16.3, 17.7)
75–84	5.9 (5.8, 5.9)	13.2 (12.9, 13.4)	14.6 (14.2, 15.1)	14.6 (14.1, 15.1)	7.1 (6.6, 7.5)
≥85	2.6 (2.6, 2.7)	4.7 (4.5, 4.8)	4.9 (4.7, 5.2)	4.8 (4.5, 5.1)	2.1 (1.8, 2.4)
Sex					
Female	54.2 (54.1, 54.3)	49.9 (49.5, 50.2)	52.6 (51.9, 53.2)	48.4 (47.7, 49.1)	48.2 (47.3, 49.1)
Male	45.7 (45.6, 45.9)	50.1 (49.8, 50.5)	47.4 (46.8, 48.1)	51.6 (50.9, 52.3)	51.8 (50.9, 52.7)
Hispanic ethnicity^b^					
Yes	18.4 (18.3, 18.5)	21.5 (21.2, 21.7)	17.7 (17.2, 18.2)	21.5 (20.9, 22.0)	29.5 (28.7, 30.3)
No	71.2 (71.1, 71.4)	74.3 (74.0, 74.6)	79.1 (78.6, 79.6)	74.8 (74.2, 75.4)	66.6 (65.7, 67.4)
Race^b^					
Asian	2.8 (2.8, 2.9)	3.2 (3.1, 3.3)	3.2 (2.9, 3.4)	3.3 (3.1, 3.6)	2.4 (2.1, 2.6)
Black	14.6 (14.5, 14.7)	24.0 (23.7, 24.2)	23.4 (22.9, 24.0)	21.0 (20.4, 21.5)	28.6 (27.8, 29.4)
White	61.8 (61.6, 61.9)	54.6 (54.2, 54.9)	59.2 (58.6, 59.8)	59.1 (58.4, 59.8)	46 (45.1, 46.8)
Other^c^	11.1 (11.1, 11.2)	13.1 (12.8, 13.3)	9.8 (9.5, 10.2)	11.3 (10.9, 11.8)	16.4 (15.8, 17.1)
BMI^b^ kg/m^2^					
<18.5	1.6 (1.6, 1.7)	1.5 (1.4, 1.5)	1.4 (1.2, 1.5)	1.3 (1.1, 1.4)	1.5 (1.3, 1.8)
18.5 to <25	21.9 (21.8, 22.0)	13.9 (13.7, 14.1)	14.1 (13.7, 14.6)	11.0 (10.6, 11.5)	12.9 (12.3, 13.5)
25 to <30	30.1 (30.0, 30.3)	25.7 (25.4, 26.0)	25.1 (24.5, 25.6)	25.5 (24.9, 26.1)	24.3 (23.5, 25.1)
30 to <35	22.8 (22.7, 22.9)	25.1 (24.8, 25.4)	25.3 (24.8, 25.8)	26.4 (25.8, 27.0)	25.3 (24.6, 26.1)
35 to <40	12.5 (12.4, 12.6)	16.6 (16.4, 16.9)	16.5 (16.0, 16.9)	18.0 (17.4, 18.5)	17.3 (16.6, 17.9)
≥40	11.0 (10.9, 11.1)	17.3 (17.0, 17.5)	17.6 (17.2, 18.1)	17.9 (17.3, 18.4)	18.7 (18.0, 19.4)
COVID-19 medications					
Dexamethasone	6.4 (6.3, 6.4)	16.5 (16.3, 16.8)	15.2 (14.8, 15.7)	15.5 (15.0, 16.0)	15.7 (15.1, 16.4)
Other corticosteroids	5.4 (5.3, 5.4)	12.6 (12.4, 12.8)	13.6 (13.2, 14.0)	11.9 (11.4, 12.3)	10.8 (10.3, 11.4)
Remdesivir	4.6 (4.5, 4.6)	13.8 (13.5, 14)	12.7 (12.3, 13.1)	14.1 (13.6, 14.5)	14.6 (14.0, 15.2)

a Includes 27,981 who met the criteria for diabetes but could not be classified by control status (i.e., did not have HbA1c measurement within 18 months before COVID-19).

b Missing data not shown.

c Other includes PCORnet values of Native Hawaiian or other Pacific Islander, American Indian or Alaska Native, multiple race, and other. DM, diabetes mellitus; PCORnet, National Patient-Centered Clinical Research Network.

## DISCUSSION

In this study of over 650,000 adults, among those with hypertension or with diabetes, severe COVID-19 outcomes were more common among those with the highest BP and HbA1c levels. Similar findings have been described in other countries; yet, this is the largest study from the U.S.^6^, ^7^, ^8^ Potential effects of poor hypertension and diabetes control on COVID-19 outcomes could disproportionately affect racial and ethnic minorities, given the longstanding disparities in chronic disease management, known health system inequities, and differential COVID-19 risk among these populations.^5^^,^^9^^,^^12^ Interestingly, the prevalence of severe COVID-19 was lower among those with BP of 130–139/80–89 mmHg than among those with BP <130/80 mmHg. Those with the lowest BP tend to be older, with more comorbidities and longer history of hypertension^13^; consistent with this pattern, persons in our analyses with BP <130/80 mmHg were older than those with BP of 130–139/80–89 mmHg.

### Limitations

A key limitation is that we could not adjust for potential confounders, so we could not explore whether differences in patient characteristics or vaccination status may partially explain the observed differences in COVID-19 outcomes by disease control status. Most of our data were collected before widespread vaccine availability, and unfortunately, most early vaccinations occurred in public health drives and were poorly captured in medical records and thus could not be assessed in this study. Other limitations include that (1) not all patients could be classified by chronic disease control status, (2) chronic disease control status may have been misclassified owing to the 18-month lookback period, and (3) approximately 10% of patients were missing data for race and 9% for ethnicity.

## CONCLUSIONS

Appropriate and equitable management of hypertension and diabetes is important,^6^, ^7^, ^8^ and the findings of this study can provide information to healthcare providers and to patients living with these chronic diseases. The COVID-19 pandemic may have disrupted healthcare visits, exacerbated existing health disparities, and adversely affected disease management.^14^ Public health messaging for patients and providers can encourage routine care, use of telehealth, and strategies such as self-measured BP monitoring and home blood glucose measurements with clinical support.^15^^,^^16^ Future work can examine whether chronic disease control status is associated with COVID-19 outcomes.
